# Total *FLC* transcript dynamics from divergent paralogue expression explains flowering diversity in *Brassica napus*

**DOI:** 10.1111/nph.17131

**Published:** 2020-12-25

**Authors:** Alexander Calderwood, Andrew Lloyd, Jo Hepworth, Eleri H. Tudor, D. Marc Jones, Shannon Woodhouse, Lorelei Bilham, Catherine Chinoy, Kevin Williams, Fiona Corke, John H. Doonan, Lars Ostergaard, Judith A. Irwin, Rachel Wells, Richard J. Morris

**Affiliations:** ^1^ Computational and Systems Biology John Innes Centre Norwich NR4 7UH UK; ^2^ Institute of Biological Environmental & Rural Sciences (IBERS) Aberystwyth University, Penglais Aberystwyth Ceredigion SY23 3DA UK; ^3^ Department of Crop Genetics John Innes Centre Norwich NR4 7UH UK; ^4^ VIB‐UGent Centre for Plant Systems Biology Technologiepark 71 Gent 9052 Belgium

**Keywords:** *Brassica napus*, flowering locus C, gene dosage balance, modelling, phenotypic plasticity, polyploidy, transcriptomics, vernalization

## Abstract

- Flowering time is a key adaptive and agronomic trait. In Arabidopsis, natural variation in expression levels of the floral repressor *FLOWERING LOCUS C* (*FLC*) leads to differences in vernalization. In *Brassica napus* there are nine copies of *FLC*. Here, we study how these multiple *FLC* paralogues determine vernalization requirement as a system.
- We collected transcriptome time series for *Brassica napus* spring, winter, semi‐winter, and Siberian kale crop types. Modelling was used to link *FLC* expression dynamics to floral response following vernalization.
- We show that relaxed selection pressure has allowed expression of *FLC* paralogues to diverge, resulting in variation of *FLC* expression during cold treatment between paralogues and accessions. We find that total *FLC* expression dynamics best explains differences in cold requirement between cultivars, rather than expression of specific *FLC* paralogues.
- The combination of multiple *FLC* paralogues with different expression dynamics leads to rich behaviour in response to cold and a wide range of vernalization requirements in *B. napus*. We find evidence for different strategies to determine the response to cold in existing winter rapeseed accessions.

## Introduction

Many plants time developmental changes to coincide with favourable seasons. To synchronize reproduction with spring, several wild and crop species require long periods of winter‐like cold to accelerate flowering, a process called vernalization. Within species, the duration of cold that plants need to be exposed to prior to flowering is a major determinant of when they flower (reviewed by Chouard, 1960). The allotetraploid crop *Brassica napus* (rapeseed, canola) has been bred for different environments in order to maximize time available for growth, reduce exposure to adverse environmental conditions and to coordinate with the land management cycle (Marjanović‐Jeromela *et al*., 2007; Canola Council of Canada, 2013). Consequently, *B. napus* varieties can be grouped into ‘crop types’. Spring types have essentially no cold requirement and are planted and harvested in the same season, with no over‐wintering. Semi‐winters have a weak vernalization requirement and are grown in regions with mild winters. Winter types are often characterized as having a strong vernalization requirement, and need to experience an extended period of cold prior to flowering (reviewed by Leijten *et al*., 2018). Within each crop type, further variation exists in the amount of cold required for flowering, and in the sensitivity of flowering time to vernalization treatment (Schiessl *et al*., 2017).

In the diploid *Arabidopsis thaliana*, most natural variation in vernalization requirement arises from differences in expression of the well‐characterized transcriptional repressor *FLOWERING LOCUS C* (*FLC*) (Johanson *et al*., 2000; Lempe *et al*., 2005; reviewed by Bloomer & Dean, 2017). FLC is a MADS‐box transcription factor that represses the central flowering regulators *FLOWERING LOCUS T* (*FT*) and *SUPPRESSOR OF OVEREXPRESSION OF CO 1* (*SOC1*) in a quantitative manner, such that in warm, long day conditions, the level of Arabidopsis *FLC* (*AtFLC*) expression is directly correlated with time to the floral transition (Lee *et al*., 2000; Michaels *et al*., 2005; Hepworth *et al*., 2020). The level of *FLC* expression is set early in seed development and is influenced both by *trans*‐factors, such as the gene *FRIGIDA,* and by noncoding *cis‐*variation at the *FLC* locus itself, to determine whether the plant will show a ‘winter‐annual’ or ‘rapid‐cycling’ type life history (Johanson *et al*., 2000; Lempe *et al*., 2005; Shindo *et al*., 2005; reviewed by Bloomer & Dean, 2017).

When Arabidopsis is subjected to winter‐like cold, *AtFLC* is transcriptionally repressed and epigenetically silenced (Sung & Amasino, 2004; reviewed by Song *et al*., 2013; Xu & Chong, 2018; Costa & Dean, 2019; He *et al*., 2020). Epigenetic silencing required the cold‐induction of VERNALIZATION INSENSITIVE3 (VIN3) a PHD‐family protein required to activate the Polycomb Repressive Complex2 at the *AtFLC* locus (Sung & Amasino, 2004). The PHD‐PRC2 complex methylates H3 lysine residues (H3K27me3) to silence transcription (Sung & Amasino, 2004; Wood *et al*., 2006; De Lucia *et al*., 2008). On return to warm conditions, *VIN3* expression is abolished, but H3K27me3 is spread across the *AtFLC* locus to maintain a stable ‘memory’ of cold, so that in spring, *AtFLC* expression remains repressed, allowing flowering to be triggered by a combination of other environmental and internal signalling pathways (Michaels & Amasino, 1999; Searle *et al*., 2006).

Epigenetic silencing at *FLC* is a slow process, occurring over weeks in the cold. H3K27me3 accumulates and represses *AtFLC* in a quantitative manner until this repression is saturated (Sheldon, 2000; Angel *et al*., 2011). The quantitative nature of *FLC* silencing and cold‐memory means that, in Arabidopsis, the flowering behaviour of natural variants is determined both by the pre‐vernalization levels of *FLC* and by the response‐rate of reduction of *AtFLC* expression in the cold (Shindo *et al*., 2006; Li *et al*., 2014; Bloomer & Dean, 2017; Hepworth *et al*., 2020).

The role of *FLC* is similar in other Brassicaceae (Leijten *et al*., 2018), however polyploids such as *B. napus* have multiple copies of genes (paralogues), many of which exhibit differences in regulation (Jones *et al*., 2018). We recently reported that flowering time genes are over‐represented in the *B. napus* genome relative to other families (Jones *et al*., 2018); in particular, *FLC* has nine identifiable copies in *B. napus*. The majority of these are believed to have been generated through whole genome duplication, as *B. napus* arose by alloploidy between the paleo‐polyploid ancestors of *B. rapa* (A‐genome) and *B. oleracea* (C‐genome), however *BnaFLC.A03b*, *BnaFLC.C03b, and BnaFLC.09a* are nonsyntenic copies, and instead arose through tandem or segmental duplication (Cai *et al*., 2014).

Over‐retention of paralogues suggests they may be dosage sensitive genes, retained to maintain stoichiometric expression balance, or alternatively, that they may play a role in facilitating adaptation and the acquisition of new functionality (Maere *et al*., 2005). A persuasive explanation for the retention of paralogues following whole genome duplication is the gene dosage hypothesis, in which gene loss would lead to negative consequences by perturbing the stoichiometric ratio of gene products. By contrast, individually duplicated dosage sensitive genes are expected to be more likely to be lost for the same reason (Birchler & Veitia, 2010, 2012). If selection acts at the level of total gene expression of a gene for which there are multiple copies, then individual paralogues can drift in their expression as long as this effect is compensated for by other paralogues (‘compensatory drift’, Thompson *et al*., 2016). Thus, within the context of a polyploid organism not all paralogous copies of a gene are necessarily equally important for its canonical function and reduced selection pressure arising from multiple copies can allow sub‐ or neo‐functionalization to a different role (Conant & Wolfe, 2008; Hua *et al*., 2009; Yu *et al*., 2018; Jones *et al*., 2018; Wu *et al*., 2019). By bringing together two paleo‐polyploid genomes with unique evolutionary histories, *B. napus* has assembled a large pool of *FLC* paralogues with potentially wide variation in expression profiles and/or function to draw upon when adapting to different flowering requirements. How, and whether all *FLC* paralogues contribute to vernalization requirement and determine flowering behaviour is therefore not clear.

There is evidence that only certain *FLC* paralogues have maintained a role in vernalization in *B. napus*. Expression of some *BnaFLC* paralogues are found to be unresponsive to cold (Schiessl *et al*., 2019). This has been interpreted to indicate that they may no longer be involved in the cold requirement machinery and may have sub‐functionalized to some different role in other processes besides cold response in flowering regulation (Schiessl *et al*., 2019). Consistent with this, associative genomics studies using different panels of accessions have identified sequence or expression variation in only a subset of paralogues (*BnaFLC.A02*, *BnaFLC.A03b*, *BnaFLC.A10*, and *BnaFLC.C02*) as associated with cold required for flowering (Hou *et al*., 2012; Wu *et al*., 2012, 2019; Raman *et al*., 2013; Song *et al*., 2020; Tudor *et al*., 2020). (See Table 1 for paralogue naming conventions.) Further evidence for the roles of specific paralogues in generating variation was provided by the demonstration that a combination of *BnaFLC.A10* with a defined transposable element together with a functional *BnaFLC.A02* is required for winter crop types (Yin *et al*., 2020). This is despite the fact that other paralogues are capable of delaying flowering in Arabidopsis, or have been implicated in flowering time control in the closely related *B. rapa* and *B. oleracea* species, suggesting that they are largely conserved in flowering‐time control (*BnaFLC.A03a*, *BraFLC.A02*, *BraFLC.A03a*, *BraFLC.A03b*, *BraFLC.A10*, *BoFLC.C02*, *BoFLC.C03b*, *BoFLC.C09a* and *BoFLC.C09b*;* *Tadege *et al*., 2001; Schranz *et al*., 2002; Pires *et al*., 2004; Lin *et al*., 2005; Kim *et al*., 2007; Razi *et al*., 2008; Irwin *et al*., 2016; Abuyusuf *et al*., 2019; Stansell *et al*., 2019).

**Table 1:** *FLOWERING LOCUS C* (*FLC*) gene identities (IDs) in *Brassica napus*.

Short *FLC* ID	Full *FLC* ID	EnsemblPlants *FLC* ID
*BnaFLC.A01*		
*BnaFLC.A02*	*BnaA02g00370D*	*GSBRNA2T00143535001*
*BnaFLC.A03a*	*BnaA03g02820D*	*GSBRNA2T00129741001*
*BnaFLC.A03b*	*BnaA03g13630D*	*GSBRNA2T00142187001*
*BnaFLC.A10*	*BnaA10g22080D*	*GSBRNA2T00135921001*
*BnaFLC.C02*	*BnaC02g00490D*	*GSBRNA2T00068991001*
*BnaFLC.C03a*	*BnaC03g04170D*	*GSBRNA2T00134620001*
*BnaFLC.C03b*	*BnaC03g16530D*	*GSBRNA2T00024568001*
*BnaFLC.C09a*	*BnaC09g46500D*	*GSBRNA2T00016124001*
*BnaFLC.C09b*	*BnaC09g46540D*	*GSBRNA2T00016119001*

Here, building on the model of gene dosage (Conant *et al*., 2014; Cheng *et al*., 2018) and drift compensation (Thompson *et al*., 2016) and extending these ideas to dynamical behaviour, we test the hypothesis that total *FLC* expression dynamics, as opposed to previously reported individual *FLC* paralogues, are key to determining flowering time. We present new transcriptome time series across a range of *B. napus* life‐histories and demonstrate that when *FLC* expression over vernalization is considered, total *BnaFLC* expression explains variation in the vernalization requirements between rapeseed crop types, suggesting that all expressed paralogues of *BnaFLC* are important in determining crop type cold requirement and response.

We show that the expression of different *BnaFLC* paralogues decline at different rates during vernalization (suggesting that the paralogues may have sub‐functionalized within their roles as quantitative environmental sensors in the vernalization pathway), resulting in expression sensitivity over different durations of cold. We find evidence that reduced selection pressure has potentiated this divergence of response.

In *B. napus*, we also see variation in the sensitivity of expression to the cold of each *BnaFLC* paralogue between accessions. Modelling suggests that this variation in cold response rate (in addition to pre‐vernalization expression levels) is important in determining vernalization requirement.

These findings suggest that total *BnaFLC* expression can be controlled by many strategies, as expression of one paralogue can be compensated for by a variety of combinations of expression of the others. Consistent with this, we see examples of numerous different *FLC* composition strategies which result in the same crop type, within the well‐studied Renewable Industrial Products from Rapeseed (RIPR) accession panel (Havlickova *et al*., 2018; Schiessl *et al*., 2019).

## Materials and Methods

### Plant growth conditions, RNA extraction, sample preparation and sequencing

*Brassica napus* cv. Stellar, Zhongshuang 11, Tapidor_JIC, Express‐617, and Ragged Jack plants were sown in cereals mix (40% medium grade peat, 40% sterilized soil, 20% horticultural grit, 1.3 kg m^–3^ PG mix 14‐16‐18 + Te base fertilizer, 1 kg m^–3^ Osmocote Mini 16‐8‐11 2 mg + Te 0.02% B, wetting agent, 3 kg m^–3^ maglime, 300 g m^–3^ Exemptor). Material was grown in a Conviron MTPS 144 controlled environment room with Valoya NS1 LED lighting (250 µmol m^−2^ s^−1^) 18°C : 15°C, day : night, 70% relative humidity with a 16 h day. At day 21, plants were put into vernalization at 5°C (8 h day).

The Tapidor accession used to generate this data is from a different seed lineage to that used in other studies (Havlickova *et al*., 2018), and during this analysis it became clear that differences exist in its *FLC* complement. Here this accession is referred to as Tapidor_JIC to differentiate it from the canonical Tapidor accession.

At each sampling timepoint three replicates samples containing either three seedings or three first true leaves were collected. For Express‐617 only one leaf sample was collected at each timepoint. Samples were ground in LN_2_ to a fine powder before RNA extraction and DNase treatment were performed following the method provided with the EZNA® Plant RNA Kit (Omega Bio‐tek Inc., http://omegabiotek.com/store/).

RNA samples were processed at Novogene (Beijing, China); complementary DNA (cDNA) libraries were constructed using NEBNext Ultra Directional Library Kit (New England Biolabs Inc., Ispwich, MA, USA), sequencing was performed using Illumina HiSeq X, resulting in 150 bp paired end reads.

### Bioinformatics

Publicly available single‐end fastq files of gene expression over vernalization were downloaded from the National Centre for Biotechnology Information (NCBI) Sequence Read Archive (SRA), project ID PRJNA398789 (Jones *et al*., 2018). Publicly available single‐end RNA sequencing data for pre‐vernalization gene expression data in the RIPR panel were downloaded from NCBI SRA, project ID PRJNA309367 (Havlickova *et al*., 2018).

Gene expression quantification was carried out using Hisat v.2.0.4 (Kim *et al*., 2015) and Stringtie v.1.2.2 (Pertea *et al*., 2015). Reads were aligned to the Darmor‐*bzh* reference genome (Chalhoub *et al*., 2014), downloaded from www.genoscope.cns.fr/brassicanapus/data/.

### Phylogenetic analyses

Coding sequences and corresponding protein sequences were curated based on aligned RNA sequencing reads, apart from *BnaFLC.C03b* which was not expressed in any sample. A codon aligned nucleotide sequence was then generated as described (Suyama *et al*., 2006) manually edited in geneious prime (v.2020.0.3). Protein sequence alignment and Neighbour‐Joining tree were generated using geneious prime with default settings. ω ratios (dN/dS) were calculated using the Ka/Ks webtool from CBU (http://services.cbu.uib.no/tools/kaks). The phylogenetic tree was plotted using the phytools R package (Revell, 2012).

### Simulation of expected RNA sequencing mis‐mapping rate between *FLC* paralogues

Using the *FLC* gene models in the Darmor‐*bzh* reference sequence (Chalhoub *et al*., 2014, http://www.genoscope.cns.fr/brassicanapus/data/), ArtificialFastqGenerator (Frampton & Houlston, 2012), was used to generate simulated reads, with read‐length, and sequencing error rates sampled from our real Fastq files. Simulated reads were put through the same alignment pipeline as the real data.

### *FLC* expression model fitting

*FLC* expression over vernalization was assumed to follow an exponential decay function. This is analogous to the equation governing radioactive decay and reflects the assumption that the probability of each *FLC* locus in a cell switching from ‘ON’ to ‘OFF’ is constant over time during vernalization.

To model experimentally measured *FLC* expression over vernalization for each paralogue of *FLC* and total *FLC* in each accession, an analysis of variance (ANOVA) test was used to select between

$$y=ae^{\mathrm{bx}}$$

which models *FLC* decay to zero given sufficient time and

$$y=ae^{\mathrm{bx}}+c$$

which models *FLC* decay to some nonzero value *c*. The selected model was carried forward for analysis. In these equations, *y* is *FLC* expression measured in TPM (transcripts per kilobase million), *x* is days of cold, and *a*, *b* and *c* are fitted parameters. The parameters *a* and c together govern pre‐vernalization expression, *b* governs the rate of decay.

For the prediction of RIPR panel *FLC* expression levels over vernalization, model 1 was assumed. The experimentally measured *FLC* expression level at 21 d was used as a, and b was set according to the assumptions described later.

## Results

### Total *FLC* expression over vernalization treatments corresponds to expected duration of cold requirement

We hypothesized that different crop types of *B. napus* will exhibit different total *FLC* expression dynamics. This was based on previous reports that link *FLC* expression to vernalization requirements in Arabidopsis accessions (Johanson *et al*., 2000; Lempe *et al*., 2005; Li *et al*, 2014; Bloomer & Dean, 2017), that flowering time depends on the decline of *FLC* expression during the cold (Shindo *et al*, 2006; Bloomer & Dean, 2017; Takada *et al*., 2019; Hepworth *et al*, 2020), and that expression levels can be tuned to be optimal for their environment (Dekel & Alon, 2005).

To test this hypothesis, we studied *BnaFLC* expression over vernalization time‐courses in six exemplar accessions of different crop types, one time‐course has been previously reported (Jones *et al*., 2018), and four were generated for this study. Stellar and Westar are spring oil seed rape (OSR) accessions, Zhongshuang 11 is a Chinese semi‐winter OSR, Tapidor_JIC and Express‐617 are winter OSR accessions, and Ragged Jack is a Siberian kale accession with a stronger vernalization requirement (Supporting Information Fig. S1).

To model the response of the *BnaFLC* copies to cold, exponential decay models were fit to the total *BnaFLC* expression data with parameters corresponding to pre‐vernalization expression levels and expression decay rates during vernalization (also referred to here as ‘cold response’). In Arabidopsis, *AtFLC* expression changes during vernalization have been described by a biphasic exponential decay model, corresponding to periods of epigenetically independent and dependent downregulation, as diagnosed by low and high expression of the PHD protein VIN3, respectively (Hepworth *et al*., 2018). However, under our experimental vernalizing conditions, *VIN3* expression rapidly increased (Fig. S2), so monophasic models were sufficient.

**Fig. 1:**
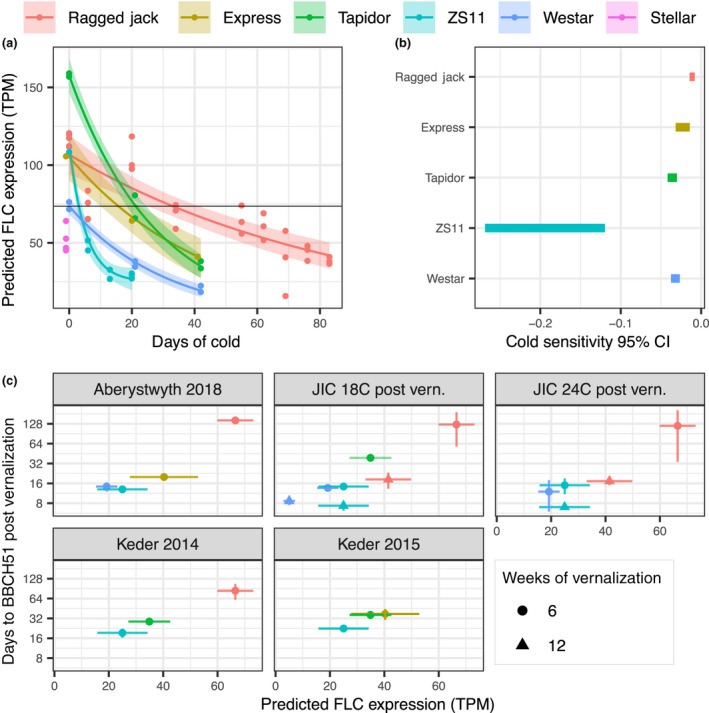
Total *FLOWERING LOCUS C* (*FLC*) expression corresponds well to vernalization requirement in *Brassica napus* when differences in expression response to cold are accounted for. (a) Pre‐vernalization *FLC* expression and response of *FLC* expression to cold are important for vernalization requirement. Time at which total *FLC* expression drops below a threshold value in each accession is consistent with cold requirement for competence to flower. Measured total *FLC* (summed expression of all nine *FLC* genes) is plotted against days of vernalization at 5°C (points). Mean and 95% confidence interval for mean plotted for each accession assuming an exponential decay model (line and ribbon). Horizontal line indicates approximate proposed floral competence threshold based on pre‐vernalization *FLC* expression in Westar. (b) The fitted *FLC* response to cold is statistically different between accessions. The 95% confidence interval range for the estimated exponential decay rate (parameter *b* in the equation $y=ae^{\mathrm{bx}}+c$; see the Materials and Methods section) of total *FLC* expression over vernalization time. (c) Below the threshold floral competence level, floral development rate corresponds well to remaining post‐vernalization total *FLC*. Predicted total *FLC* expression is derived from models fit to *FLC* expression over vernalization for periods of 12 wk (Ragged Jack), 6 wk (Tapidor_JIC, Westar), or 3 wk (Zhongshuang 11) as shown in (a). The number of days post‐vernalization to reach developmental stage BBCH51 (buds visible, Meier *et al*., 2009) were measured in separate experiments after 6 or 12 wk of vernalization. Mean values and 95% confidence intervals for mean values are plotted. The association between predicted *FLC* and time taken is surprisingly clear, considering that different individuals are considered, and for some cases the extrapolation of vernalization times from the data used to fit the models. This is most extreme for Zhongshuang 11 (ZS11), in which final measurements at 3 wk are used to make predictions at 12 wk, which may explain its relatively poor agreement with the consensus pattern. Expression levels are given in TPM (transcripts per kilobase million).

**Fig. 2:**
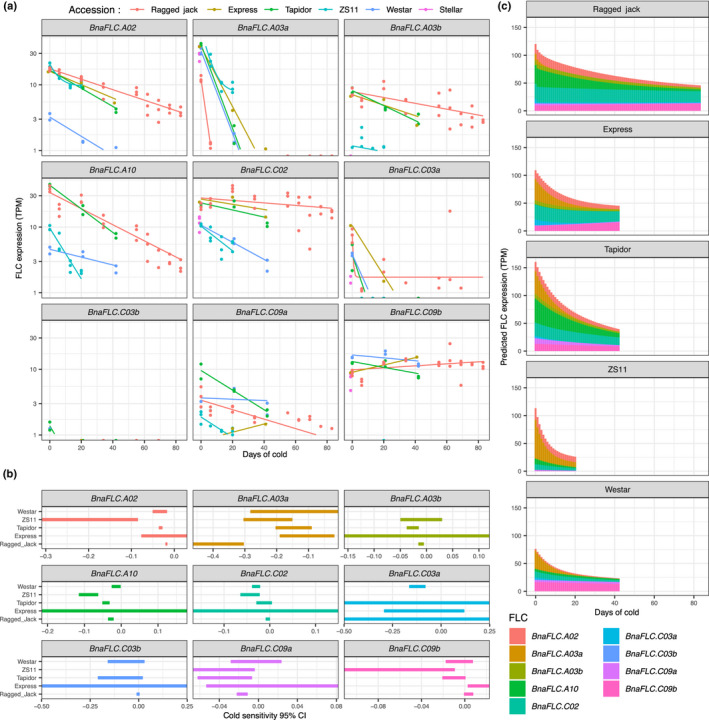
Differences in response of total *FLOWERING LOCUS C* (*FLC*) expression during vernalization are a consequence of pre‐vernalization composition of *FLC* paralogues, and of differences in the cold response of the same loci between *Brassica napus* accessions. (a) Experimental and fitted models for expression of individual *FLC* paralogues over vernalization. The *Y*‐axis is log scale, meaning that the gradient of the lines is equal to the exponential decay rate b. Differences in this rate are clearly seen between the major expressed paralogues. (b) Confidence intervals show that differences between paralogues, and between accessions at a single locus can be statistically significant. The 95% confidence interval range for the estimated decay rates in the different *FLC* paralogues among the six‐accession panel. (c) Predicted expression levels of individual *FLC* paralogues, stacked to show contribution to total *FLC* level in different accessions. Variation in the relative expression of cold responsive and unresponsive paralogues, as well as cold response at each locus contribute to the quantitatively different behaviour of total *FLC* over cold. Expression levels are given in TPM (transcripts per kilobase million).

As shown in Fig. 1(a), pre‐vernalization total *BnaFLC* expression does not correspond well to vernalization requirement. For example, the winter variety Tapidor_JIC has higher pre‐vernalization total *BnaFLC* expression than Ragged Jack but requires a shorter vernalization period (Fig. S1). Furthermore, the semi‐winter Zhongshuang 11 has similar pre‐vernalization *BnaFLC* expression levels to the winter type accessions Express‐617 and Ragged Jack. However, there are clear differences in the rate at which total *BnaFLC* expression declines under vernalization between varieties. For example, the initially high levels of *BnaFLC* seen in Zhongshuang 11 decay very rapidly under these vernalizing conditions. These differences are statistically significant given the exponential decay assumption (Fig. 2b).

The spring variety Westar flowers rapidly without cold treatment. If pre‐vernalization total *BnaFLC* expression in the spring variety Westar is taken as the approximate level which is insufficient to prevent flowering, we find that the order in which the total *BnaFLC* of each variety is predicted to cross this threshold corresponds to their vernalization requirement based on crop type. Use of this threshold level is consistent with the experimental observation that Ragged Jack requires *c.* 6 wk (42 d) vernalization at 5°C for floral competence (Fig. S1). Below this threshold, predicted total *BnaFLC* expression levels after 6 and 12 wk vernalization correspond well to the time taken to reach BBCH51developmental stage (buds visible, Meier *et al*., 2009) after removal from vernalizing conditions (Fig. 1c). We conclude that total *BnaFLC* expression dynamics correlate with different cold requirements between *B. napus* crop types.

### Differences in total *BnaFLC* cold response are caused both by differences between paralogues within an accession and differences in homologue behaviour between accessions

We next asked what might cause the different rates of decline in total *BnaFLC* expression. We hypothesized that different *FLC* paralogues may have different expression decay rates. To investigate this, we examined the expression of the nine individual paralogues in each *B. napus* accession.

As different *BnaFLC* paralogues are highly similar, being up to 98.48% identical at the sequence level (*BnaFLC.A03a* vs *BnaFLC.C03a*), we first ensured that our RNA sequencing data was able to distinguish between the paralogues. Under simulation, paired‐end reads are able to distinguish well between paralogues (Fig. S3; Table S1). For shorter, single‐end RNA sequencing data (Havlickova *et al*., 2018; Jones *et al*., 2018), more mis‐mapping can be expected, but we see that paralogues can still be distinguished (Fig. S4).

As previously reported, some paralogues are expressed at different levels in the different accessions prior to vernalization (Schiessl *et al*., 2019). Interestingly, Express‐617 (a winter type accession), has spring‐type levels of pre‐vernalization *BnaFLC.A10* (Fig. 2a), which has been repeatedly identified as discriminating between spring and winter behaviour (Hou *et al*., 2012; Schiessl *et al*., 2017, 2019; Wu *et al*., 2019; Song *et al*., 2020). However, in Express‐617 this spring‐type *BnaFLC.A10* paralogue is compensated for by higher expression of a combination of other *BnaFLC* paralogues, notably *BnaFLC.A02*, *BnaFLC.C02* and *BnaFLC.C03a*, resulting in a high total level of *BnaFLC*. This finding emphasizes the importance of considering the *BnaFLC* loci in combination, rather than individually.

In addition to differences in pre‐vernalization expression level, statistically significant differences in cold expression response exist between paralogues (Fig. 2a,b). For example, the expression of *BnaFLC.A03a* paralogue declines rapidly with time across all tested accessions in the first 3 wk of vernalization (strong cold response). By contrast, expression of the *BnaFLC.C09b* locus either maintains, or slightly increases expression during cold in all accessions (cold insensitive). Other paralogues have varied cold responses between these two extremes. Interestingly, there are also significant differences in the rate at which expression declines between accessions for equivalent *FLC* paralogues, including in the *BnaFLC.A02*, *BnaFLC.A10* and *BnaFLC.C02* copies, genes which have recently been associated with crop type differences (Song *et al*., 2020; Yin *et al*., 2020).

In order to assess the relative importance of the pre‐vernalization *BnaFLC* composition of highly cold responsive and unresponsive *BnaFLC* paralogues, as compared to differences in responsiveness at the same loci between accessions, we plotted how the individual paralogues are predicted to contribute to the total *BnaFLC* level over cold days (Fig. 2c). This analysis suggests that Zhongshuang 11 has a low vernalization requirement compared to winter crop types primarily because the rapid‐vernalizing *BnaFLC.A03a* type makes up a large proportion of its total pre‐vernalization *FLC*. Although expression of many other paralogues decays rapidly in Zhongshuang 11 relative to other accessions, they are not initially expressed at a high level, so that their large cold response has relatively little effect on total *BnaFLC* response. By contrast, Ragged Jack appears to require an extreme vernalization treatment relative to Tapidor_JIC and Express‐617, partly because its pre‐vernalization *FLC* composition is weighted away from *BnaFLC.A03a*, towards more stable paralogues, but also because many paralogues of *FLC* appear to be less cold responsive than in Express‐617 or Tapidor_JIC. For example, *BnaFLC.A10* is highly expressed in both Tapidor_JIC and Ragged Jack, but is more stably expressed in the latter.

Together, these analyses suggest that total *FLC* expression dynamics, comprising individual *FLC* paralogues that have diverged in their response to cold, determine vernalization requirements in the six studied *B. napus* accessions.

### Divergent expression dynamics are associated with relaxed selection strength acting on coding sequences

**Fig. 3:**
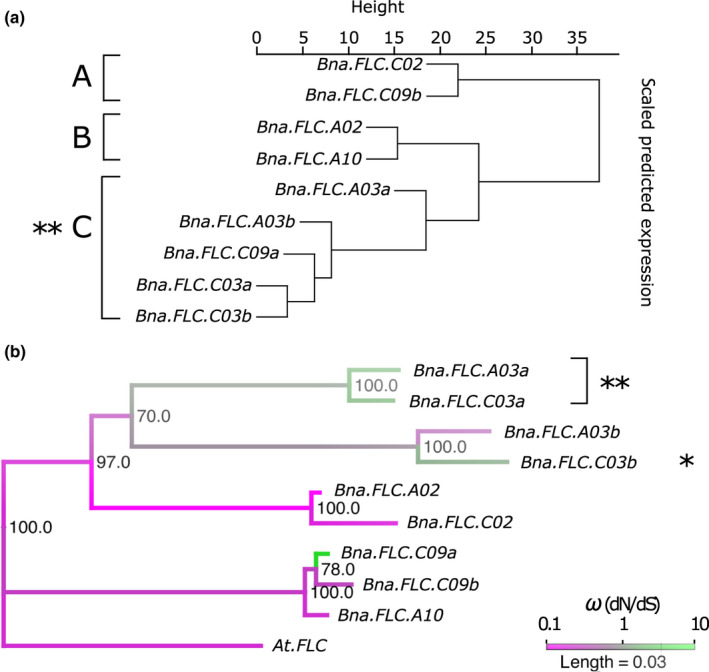
Divergent expression dynamics are associated with relaxed selection on coding sequences. (a) *Brassica napus FLOWERING LOCUS C* (*FLC*) genes clustered by similarity in expression during vernalization. Dendrogram shows clustering by Euclidean distance between expression profiles during vernalization in the six core accessions. Three main expression types are observed (A, B, and C) corresponding to cold stable expression (A), moderate decay rate through vernalization (B) and rapid decay or low expression levels (C). (b) A phylogenetic tree based on codon aligned coding sequences with Arabidopsis *FLC* (*AtFLC*) as an outgroup. Bootstrap confidence levels (percentage of 1000 replicates) are indicated at each node. Colour scale indicates the ω ratio calculated along each branch. Asterisks indicate the significance levels of the Relax test for relaxed selection (*,*P* < 0.05; **,*P* < 0.01).

In order to gain insights into the diversity of expression dynamics observed for the multiple *BnaFLC* paralogues, as well as the origin of this variation, we first clustered the *BnaFLC* genes based on their expression dynamics in the six accessions (Fig. 3a). This clustering identified three main expression types (A, B and C). Cluster A comprised genes with relatively cold stable expression (*BnaFLC.C02*, *BnaFLC.C09b*). Cluster B comprised genes with a moderate expression decay rate (*BnaFLC.A10*, *BnaFLC.A02*) and cluster C consisted of genes that had either a rapid decay rate or low overall levels of expression (*BnaFLC.A03a*, *BnaFLC.A03b*, *BnaFLC.C03a*, *BnaFLC.C03b*, *BnaFLC.C09a*). Notably, this clade included all genes that arose through tandem or segmental duplications (*BnaFLC.A03b*, *BnaFLC.C03b*, *BnaFLC.C09a*), including the presumed pseudogene (*BnaFLC.C03b*).

The gene balance hypothesis (Birchler & Veitia, 2010) predicts that duplicates of dosage sensitive genes are less likely to be selectively maintained following segmental duplication than following whole genome duplication (Conant *et al*., 2014; Cheng *et al*., 2018). We wondered whether the divergent expression types seen for type C genes might therefore be associated with altered signatures of selection.

To investigate this further we determined the ratio of nonsynonymous to synonymous substitution rates (ω = dN/dS) along branches of an *FLC* phylogenetic tree. All genes belonging to expression types A and B showed ω values below 0.5 consistent with purifying selection (Fig. 3a), i.e. selection against deleterious variation.

By contrast, four out of five type C expression genes had ω values at or above 1, consistent with relaxed or positive selection (Fig. 3b). We subsequently used Relax, a test based on random effects branch‐site evolutionary models (Wertheim *et al*., 2014), to formally compare the strength of selection acting on expression type C genes, to that acting on type A and B genes. Rather than use a single gene‐wide ω, Relax estimates a distribution of ω values over all sites. Importantly, this allows it to efficiently distinguish between an increase in ω due to general relaxed selection from an increase in ω due to intensified positive selection at a sub‐set of sites. Explicitly, Relax estimates a separate discrete distribution of ω for reference and test branches in a codon‐based phylogenetic framework. A likelihood ratio test then compares the null model in which the distribution of ω is the same for reference and test branches to an alternative model in which the test ω distribution is modified by a free ‘selection intensity’ parameter *K*. A value of *K* < 1 indicates relaxed selection whilst a value of *K* > 1 indicates increased selection (Wertheim *et al*., 2014).

The Relax test demonstrated that coding sequences of type C genes have experienced a significant reduction in selection strength compared to those of expression type A and B (*K* = 0.03, *P* = 0.0017). We re‐ran the analyses for individual genes or pairs of genes with similar expression profiles in clade C. Relaxed selection was confirmed for *BnaFLC.A03a*/*BnaFLC.C03a* (*K* = 0.00, *P* = 0.0012) and for the pseudogene *BnaFLC.C03b* (*K* = 0.00, *P* = 0.045) but not for *BnaFLC.A03b* (*P* = 0.641) suggesting that this last gene is still experiencing purifying selection, consistent with its lower ω value (Fig. 3b). *BnaFLC.C09a* is a very recent duplicate and had therefore accumulated insufficient substitutions (a single nonsynonymous substitution) for meaningful analysis. These findings are consistent with the hypothesis that individual paralogues may drift in their expression and that reduced expression may reduce selection pressure and promote neo‐ and sub‐functionalization (Thompson *et al*., 2016). In the case of *BnaFLC.A03a*, and *BnaFLC.C03a*, this sub‐functionalization apparently takes the form of an increased cold response sensitivity, diversifying the range of cold response rates available to the polyploid within the *FLC* gene group.

### Different strategies for pre‐vernalization *BnaFLC* composition exist within the winter crop type

The finding that total *FLC* dynamics is determined by combining paralogues with different cold responses suggests that different combinations could be employed to achieve a similar outcome. In order to assess this inference we considered expression of *BnaFLC* paralogues in a subset of the RIPR panel comprising 37 spring, 43 winter, six semi‐winter crop types, and nine swede types (Havlickova *et al*., 2018). The RIPR panel data was collected pre‐vernalization (Havlickova *et al*., 2018). This panel includes the six varieties measured in our time‐course over vernalization.

**Fig. 4:**
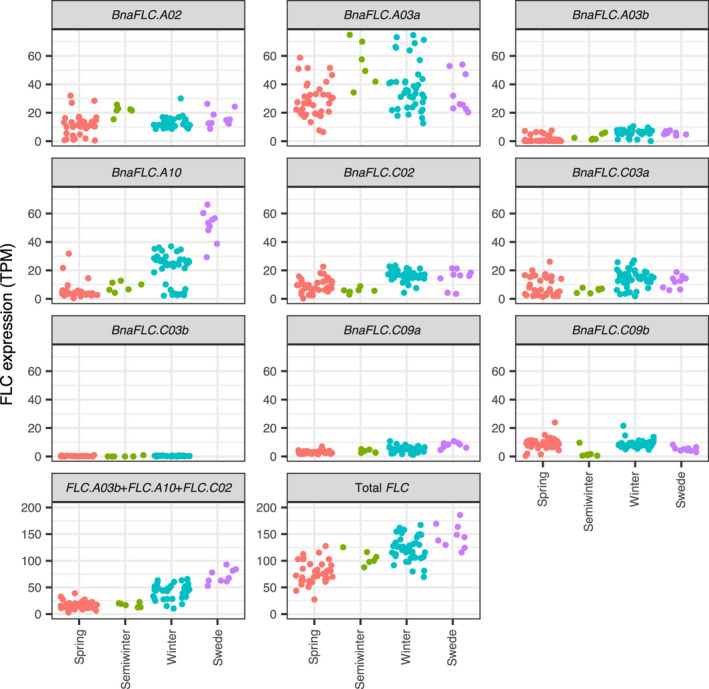
Pre‐vernalization *FLOWERING LOCUS C* (*FLC*) levels in *Brassica napus* do not distinguish well between crop types. Each point corresponds to *FLC* expression in a 3‐wk‐old un‐vernalized leaf sample. Although total *FLC* expression is associated with crop type (as there is a difference in the mean expression level between spring and winter types), it does not distinguish between crop types (as the distributions of expression levels in spring and winter crop types overlap), and so it is not sufficient to explain why an accession has a spring type or a winter type vernalization requirement. Similarly, expression of some individual paralogues (*BnaFLC.A03b*, *BnaFLC.A10*, *BnaFLC.C02*) were found to associate with the spring, winter split (Schiessl *et al*., 2019), but are not sufficient to separate them, either considered individually or in combination (*BnaFLC.A03b* + *BnaFLC.A10* + *BnaFLC.C02*). Expression levels are given in TPM (transcripts per kilobase million).

Consistently among the semi‐winter varieties included in the RIPR panel, a comparatively large proportion of the total *BnaFLC* is made up of the highly responsive *BnaFLC.A03a* copy (Fig. 4). This fits with a simple hypothesis, in which prior to vernalization the *BnaFLC.A03a* copy inhibits flowering, but rapidly decays with cold, leaving the total other paralogues insufficiently expressed to prevent flowering. Conversely the swede types, which have an extreme, long vernalization requirement, commonly have a low *BnaFLC.A03a* to *BnaFLC.A10* expression ratio relative to other accessions.

As previously reported, although total *BnaFLC* expression prior to vernalization is associated with crop type, it is a poor discriminant between spring and winter types in this panel (Fig. 4, total FLC subplot), and pre‐vernalization expression levels of *BnaFLC.A10*, *BnaFLC.A03b* and *BnaFLC.C02* provide the best discriminants between spring and winter crop types in this data (Schiessl *et al*., 2019). However, pre‐vernalization *FLC* expression in the RIPR panel makes it clear that Express‐617 is not unusual in having atypical expression of one of these key paralogues for its crop type. Fig. 4 shows that expression of these paralogues is insufficient to explain the spring–winter crop type split: the range of expression levels of every individual paralogue of *BnaFLC* shows a large overlap between spring and winter crop types. Of the 37 spring and 43 winter accessions, eight spring types and four winter types have noncanonical expression of *BnaFLC.A03b*. Three spring accessions, and 11 winter accessions have noncanonical *BnaFLC.A10* expression levels, and the majority of spring types have greater *BnaFLC.C02* expression than the lowest winter type.

It is therefore insufficient to consider each *FLC* paralogue in isolation to explain the cold requirements of these accessions. Moreover, a simple combination of pre‐vernalization expression of the paralogues which have previously been identified as best distinguishing spring from winter are still not able to separate them. Considering *BnaFLC.A03b* + *BnaFLC.A10* + *BnaFLC.C02*; 34 spring lines express more of these than the lowest winter line, and 12 winter lines express less than the highest spring line.

**Fig. 5:**
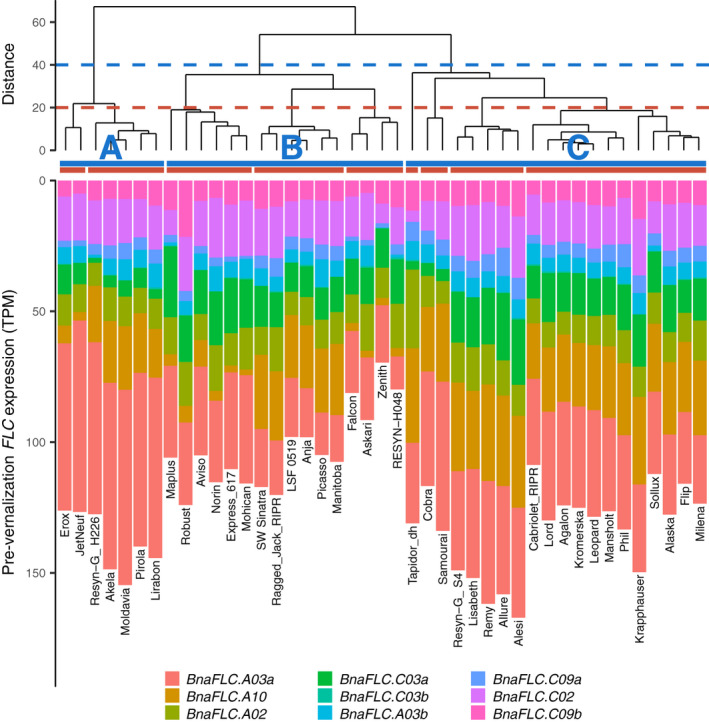
Different *FLOWERING LOCUS C* (*FLC*) composition strategies exist among *Brassica napus* winter crop type accessions. Pre‐vernalization *FLC* composition of winter crop type accessions, clustered by similarity in *FLC* paralogue expression. Dendrogram shows clustering by Euclidean distance between pre‐vernalization *FLC* expression in each accession. Horizontal bars show cluster membership if cut at height of 40 (blue), or 20 (red). Bar‐plot shows pre‐vernalization *FLC* expression, *FLC* paralogues are ordered by approximate cold stability from most stable (*BnaFLC.C09b*) to least (*BnaFLC.A03a*). There are three broad *FLC* compositions (A, B and C groups) within winter type accessions. The B group has low total expression, but proportionately more stable paralogue expression. The A and C groups have higher total FLC expression, the A group has high expression of the rapidly decaying *BnaFLC.A03a* copy. Within these groups, further subtypes exist (see e.g. red clusters). For example; the A group is further split by whether *BnaFLC.A10* is expressed. Expression levels are given in TPM (transcripts per kilobase million).

Among the winter‐type accessions, several different ‘strategies’ for the composition of pre‐vernalization *FLC* have arisen (Fig. 5). Broadly, there are two different high pre‐vernalization total *FLC* strategies (groups A and C), and one lower total *FLC* group (group B). Members of group B express proportionately more of the cold‐stable *FLC* paralogues identified in the previous section, whereas for example in group A, approximately half of the total *FLC* is composed of the rapidly cold responsive *BnaFLC.A03a* copy. Further nested subgroups are evident within each of these broad strategies. For example; within both groups A and B, there are examples of spring and winter type *BnaFLC.A10* expression, which are compensated for by expression levels of various other paralogues.

These results confirm that high pre‐vernalization levels of total *FLC* expression do not imply a strong vernalization requirement, and importantly show that different combinations of paralogue expression are employed to achieve the same (winter) crop type. The *FLC* composition strategies seen among winter accessions suggest that paralogue response to cold, as well as pre‐vernalization expression level should be considered.

### Total *BnaFLC* explains cold requirement in the RIPR diversity panel if differences in the response of *FLC* expression to cold are considered

**Fig. 6:**
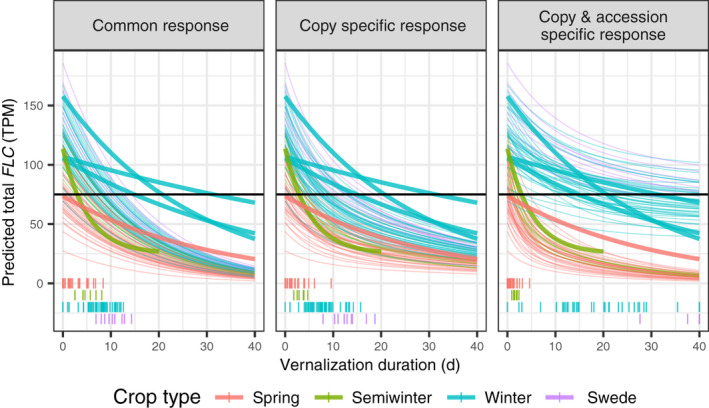
Difference in decay rate between *Brassica napus* accessions is required to achieve realistic vernalization predictions. The predicted *FLOWERING LOCUS C* (*FLC*) expression levels over vernalization for Renewable Industrial Products from Rapeseed (RIPR) panel accessions. A threshold *FLC* expression for floral competence is shown at 75 TPM (transcripts per kilobase million). Bold lines show modelled *FLC* expression over vernalization for our six‐accession panel, based on measured expression (also shown in Fig. 1a). The time at which each accession crosses the floral competence threshold is shown with vertical dashes. A threshold of 75 TPM is used as this is the measured pre‐vernalization expression level of Westar, which has no vernalization requirement, and is taken as an approximation of the required level to be able to flower upon cessation of cold treatment. Day 0 expression is taken from measured expression in the leaf at 21 d. Decay parameters are based on values measured in six accessions, under different assumptions; (left) common response; all decay rates are the same for all paralogues in all accessions, (middle) paralogue specific response; *FLC* paralogues have different cold response rates, which is set to the mean measured value or each paralogue, (right) paralogue and accession specific response; the same paralogue of *FLC* may have different decay rates in different accessions. The decay rate for each paralogue in each accession is set to the most extreme maximum‐likelihood value estimated for that *FLC* paralogue among the six‐accession panel, in order to maximize the difference between the low vernalization (spring and semi‐winter) and high vernalization (winter and swede) groups. Only by accounting for differences in cold response between paralogues and accessions is good separation of crop types and realistic vernalization requirements for winter crop types predicted (vertical dashes).

Pre‐vernalization *FLC* expression is only weakly associated with length of cold requirement. To assess whether differences in the cold response of *BnaFLC* expression are sufficient to explain variation in cold requirement between crop types, we modelled the predicted level of *BnaFLC* expressed in the RIPR lines during vernalization treatment (Fig. 6). We used the experimentally measured pre‐vernalization *BnaFLC* expression levels as a starting level for each accession. The exponential decay rate parameter was set based on the values previously measured in the six accessions, under three different assumptions. It was either (1) set as the mean measured cold response value for all paralogues of *BnaFLC*, so assuming no differences in decay rate between paralogues or accessions (Fig. 6, left**)**. Or (2) set to the mean value across accessions for each individual paralogue, meaning for example that *BnaFLC.A03a* decays relatively rapidly, and so assuming no differences between accessions (Fig. 6, middle). Or (3) set to the most extreme measured response for each paralogue among the six accession panels, so as to best separate spring and semi‐winter (rapid cold response) from winter and swede crop types (slow cold response), this allows for differences in decay rate between *FLC* paralogues, and between accessions (Fig. 6, right). In each panel, bold lines show the predicted values using the accession models fit to measured *BnaFLC* levels during vernalization as previously discussed (see Fig. 1a). Vertical dashes indicate when each accession crosses below the Westar‐derived total *FLC* threshold level and are predicted to be competent to flower.

If differences in cold response between paralogues are not taken into account (Fig 6, left), then separation in predictions for required cold treatment between crop types is poor, reflecting the low association of pre‐vernalization total *BnaFLC* with crop type and its poor predictive power.

If different *BnaFLC* paralogues are allowed different decay rates (Fig 6, middle), the results are slightly better in terms of crop type separation, reflecting the fact that differences in pre‐vernalization *BnaFLC* composition between accessions contribute to differences in their cold requirement. However, this simple model predicts unrealistically short cold requirements in winter and swede varieties, which often require more than 12 wk vernalization (Schiessl *et al*., 2017).

To separate spring and winter crop types, and predict realistic cold period requirements for winter accessions, differences in the cold response of individual paralogues between accessions must be considered (Fig 6, right). Under this assumption, three winter types (Falcon, Resyn‐H048, Zenith) are predicted to require less cold than the most cold‐dependent spring type. This is also the only tested assumption under which measured expression in the winter accessions Express‐617 and Tapidor_JIC is not misrepresentative of the predicted *BnaFLC* expression for winter accessions in the RIPR panel.

More detailed predictions of the vernalization requirements for these accessions are not possible without further characterization of *FLC* response to cold in them. However, this approach successfully demonstrates that differences in cold response of *BnaFLC* expression within the range observed in a small panel of accessions are sufficient to largely explain crop type differences in this broader group.

Taken together, our data, analysis and modelling support the view that total *BnaFLC* expression dynamics rather than individual paralogues are the key factor determining vernalization requirements.

## Discussion

The allotetraploid crop *B. napus* has nine copies of *FLC*, a key gene that determines a plant’s vernalization requirement and associates with adaptation to different agricultural environments by adjusting flowering time. Individual *FLC* paralogues have been linked to different *B. napus* crop types (Hou *et al*., 2012; Wu *et al*., 2012, 2019; Raman *et al*., 2013; Schiessl *et al*., 2019; Song *et al*., 2020; Yin *et al*., 2020); yet there are discrepancies between observed and expected *FLC* expression levels based on a crop type classification of accessions; and transferring seemingly specific crop type‐defining paralogues into a different crop type did not resulted in the expected changes (Yin *et al*., 2020).

**Fig. 7:**
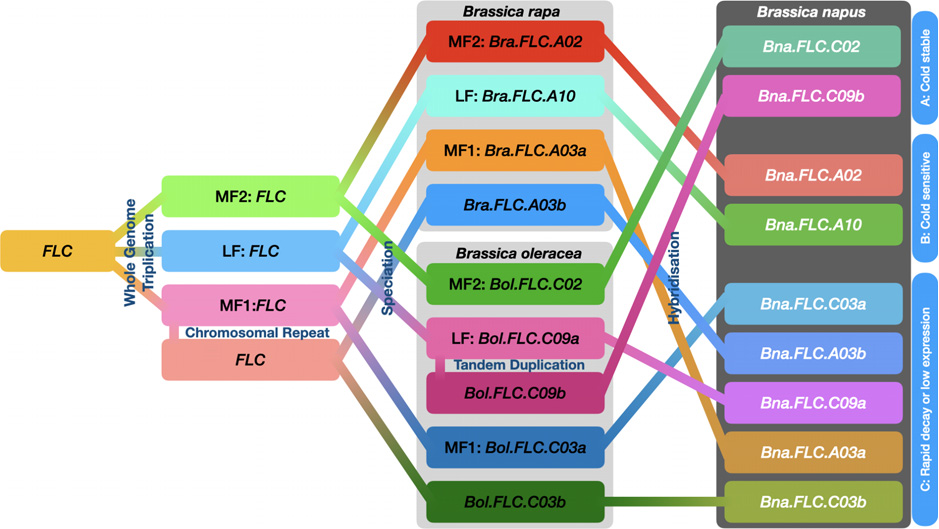
Evolutionary relationship between *Brassica napus FLOWERING LOCUS C* (*FLC*) paralogues and their cold response dynamics. Evolutionary path of *FLC* in *Brassica napus* following Schiessl *et al*. (2019), starting from a genome wide triplication in an ancestor with a single *FLC* copy (related to Arabidopsis). A subsequent gene duplication event (chromosomal repeat and inversion) is thought to predate the speciation into *B. rapa*, *B. oleracea* and *B. nigra* (*B. nigra* not shown here.) A further gene duplication (tandem duplication) event took place within the *B. oleracea* lineage. All nine *FLC* paralogues in *B. napus* (hybridization between *B. rapa* and *B. oleracea*) have been retained and have developed different cold response dynamics, possibly as a consequence of gene dosage balance acting on total *FLC* and drift compensation.

Here, we investigated how multiple paralogues of the flowering gene *FLC* may function as a system to determine vernalization requirements in *B. napus*. In contrast to previous studies (Schiessl *et al*., 2019), which have largely focused on pre‐vernalization *FLC* expression, we demonstrate that when *BnaFLC* expression response to cold is additionally considered, total *BnaFLC* expression can explain cold requirement in *B. napus* better than individual ‘important’ paralogues. The importance of total *BnaFLC* suggests that all copies are relevant in determining vernalization requirement, including copies which are themselves cold‐unresponsive, Fig. 7.

We suggest that differences between accessions in the response of total *BnaFLC* expression to vernalization are controlled both by varying the pre‐vernalization composition of expressed *BnaFLC* paralogues (with different cold responses), as well as through differences in the cold responses of *BnaFLC* orthologues between accessions. This parallels the case in Arabidopsis, in which variation between accessions in both pre‐vernalization expression and cold‐downregulations at the single *AtFLC* locus are important for the local adaptation of accessions (Lempe *et al*., 2005; Li *et al*., 2014; Duncan *et al*., 2015; Méndez‐Vigo *et al*., 2016; Bloomer & Dean, 2017; Whittaker & Dean, 2017). In *B. napus,* this variation is spread across multiple paralogues within a single species. How then does such wide variation in the expression dynamics of different paralogues arise? One example is provided by the drift compensation model (Thompson *et al*., 2016) which demonstrates how the expression of individual paralogues can move freely so long as other paralogues compensate for any introduced expression changes. One prediction of this model is that over time the expression level of some genes will reduce sufficiently that selection is relaxed, allowing the evolution of new function, new expression dynamics, or pseudogenization. Consistent with this model, *BnaFLC* paralogues with low levels of expression such as *BnaFLC.C03b* or novel dynamics, such as the highly cold responsive paralogue *BnaFLC.A03a* show evidence of relaxed selection.

It is also possible for variation to arise in the context of purifying selection. Of the paralogues that have *ω* < 0.5, those belonging to the C genome form one expression type (cold‐stable), while those belonging to the A genome form another (cold responsive). This is despite each A genome paralogue being more closely related phylogenetically to its direct C genome homoeologue (Fig. 3). This suggests that differential selection in the *c.* 2.5–4.5 Myr since divergence of the A and C genomes (Arias *et al*., 2014; Liu *et al*., 2014) but prior to the formation of *B. napus* may also have contributed to variation in expression dynamics. Thus, compensatory drift, relaxation of selection and differential selection all have the potential to generate diversity in expression dynamics which, when brought together in a single species provides a pool of variation that may be more rapidly evolvable than a single gene.

An example of how altered expression levels contribute to the rapid evolution of cold sensitivity within the context of *BnaFLC* can be seen in the Chinese semi‐winters (Fig. 4). Since the introduction of winter accessions to China in the 1940s, selective breeding for a milder cold requirement has apparently led to an increase in the relative pre‐vernalization expression of the highly cold responsive *BnaFLC.A03a* paralogues, and decrease in the expression of cold stable paralogues, resulting in a dramatically different expression of total *BnaFLC* over vernalization, and consequently a novel vernalization response phenotype, distinct from both spring and winter crop types.

Based on the hypothesis that total *FLC* is important, we might expect to find different ways of implementing the same total *BnaFLC* behaviour through different combinations of paralogue dynamics. Consistent with this idea, within the winter crop type, we observe a number of different strategies for managing total *BnaFLC* through variation in the pre‐vernalization *BnaFLC* composition of differently cold responsive paralogues. A consequence of this diversity of viable strategies is that association studies are more likely to be sensitive to the composition of the accessions studied, than for phenotypes where the strategy space is smaller. When paralogues can compensate for each other and produce the same phenotype, it is not clear whether a statistical association between a trait and a particular paralogue means that it is truly contributing more to producing a phenotype within an individual, or is instead associated with a more common strategy within the panel. Here, cold requirement provides a case study for the additional difficulties of gene association studies with polygenic traits.

Although we find that separation between the cold requirement of spring and winter crop types is much better after consideration of differences in the cold response of gene expression, the model is not perfect. For example, three winter accessions (out of 43) are not predicted to require more vernalization than the most cold‐requiring spring type. This is likely to be caused by a combination of several factors (1–4). (1) Although the ASSYST crop type labels (spring, semi‐winter, winter, swede) are a useful shorthand for vernalization phenotype, they are not perfect; there is variation in cold requirement with each crop type group, and the labels can be misleading. For example, the winter variety Mansholt has been reported to flower without vernalization, whereas the spring varieties Giant‐xr707 and Daichousen (fuku) do require vernalization (Schiessl *et al*., 2017). (2) The hard *FLC* expression threshold for floral competence considered here is an approximation and a simplification. The level used was based on the pre‐vernalization *BnaFLC* expression of the spring variety Westar, and thus was considered to provide a lower bound on the true threshold level. However, any ‘threshold level’ is also likely to vary between accessions, partly as a consequence of the next point. (3) Variation at genes downstream, or independent of *FLC* expression in the vernalization pathways can also affect cold requirement. For example, the low vernalization requirement in Mansholt has been linked to sequence variation in the promoter of *BnaFT.C02* (Schiessl *et al*., 2017). *BnaFT.A02*, *BnaFT.C06a* and *BnaFT.C06b* have also been associated with two major QTL clusters for flowering time (Wang *et al*., 2009; Tudor *et al*., 2020), and may modify the effects of *FLC*. (4) The range of decay parameters allowed for each *FLC* paralogue were derived from measurements of only six accessions, and it is likely that more extreme *FLC* paralogue responses to cold exist within the RIPR panel.

Differences in the cold sensitivity of expression between paralogues suggest that, as in Arabidopsis (Shindo *et al*, 2006; Li *et al*., 2014; Bloomer & Dean, 2017), cold sensitivity is at least partially a consequence of *cis* sequence differences at the *BnaFLC* loci. However, we also see common patterns across paralogues within accessions (for example many paralogues have relatively stable expression in Ragged Jack). This suggests that functionally important differences in responsiveness are also caused by trans factors, which regulate multiple *BnaFLC* loci during vernalization. Consistent with this, genes involved in chromatin modification at *FLC* have experienced selection during crop type improvement (Lu *et al*., 2019). More thoroughly studying the range of cold responses in a broad panel of accessions is therefore an important next step in order to understand this system.

In summary, our findings support the idea that total *BnaFLC* expression, rather than individual *BnaFLC* paralogues, determine vernalization requirement in *B. napus*. Central to this proposition is consideration of the dynamic cold response of *BnaFLC* expression, which we modelled using an exponential decay function. The proposed hypothesis provides a simple, mechanistically‐based explanation of an accession’s cold requirement, and thus is a useful framework from which to further study vernalization in this polyploid system. Given information on pre‐vernalization expression levels, the current model allows for simple predictions of the magnitude of vernalization requirement attributable to each *FLC* copy in a given accession. As more data regarding the quantitative response of paralogues to cold becomes available, we hope to be able to produce more refined predictions for the duration of vernalization necessitated by different *FLC* complements.

The importance of combined *BnaFLC* levels suggests that *B. napus* may have retained so many *FLC* paralogues, because (in the absence of significant feedback regulation) loss of a duplicate results in a quantitative difference in total *BnaFLC* expression level, which may be sufficient to lead to detrimental changes in phenotype. Notably, this is the case even for paralogues which are themselves unresponsive to vernalization. Such considerations are consistent with gene‐dosage selection (Conant *et al*., 2014) and drift compensation (Thompson *et al*., 2016) applied to expression dynamics, and suggest a means by which paralogue combinatorics could be exploited to potentiate phenotypic diversity in polyploids.

## Author contributions

AC designed and performed the majority of the data analysis. AC, JH, RJM and AL wrote the manuscript with contributions from all co‐authors. AL performed the phylogenetic analysis and selection pressure calculations. JH, EHT, DMJ, SW, LB, FC, KW, CC, JAI and RW performed the experiments. RJM, RW, LO, JHD and JAI supervised the project.

## Supporting information


**Fig. S1** Ragged Jack is an extreme winter type relative to Tapidor_JIC and Express‐617.
**Fig. S2** VIN3 is rapidly induced, meaning that epigenetic dependent and independent vernalization periods are not distinguishable under our experimental conditions.
**Fig. S3** *FLC* gene sequences are sufficiently different that RNA sequencing can distinguish them.
**Fig. S4** *FLC* gene sequences for publicly available 100 bp single‐end RNA reads are sufficiently different that RNA sequencing can distinguish them.

**Table S1** Only minor divergence from the reference sequence is observed in the *FLC* coding sequence.
Please note: Wiley Blackwell are not responsible for the content or functionality of any Supporting Information supplied by the authors. Any queries (other than missing material) should be directed to the *New Phytologist* Central Office.

## Data Availability

The data that support the findings will be available in SRA, BioProject ID PRJNA648031, following an embargo from the date of publication to allow for commercialization of research findings.
