# Supplementary material for: Total FLC transcript dynamics from divergent paralogue expression explains flowering diversity in Brassica napus
Source: New Phytol. 2020 Dec 25;229(6):3534–48. doi: 10.1111/nph.17131 (PMC7986421; doi:10.1111/nph.17131)
Supplement: Supplementary file 1 — Fig. S1 Ragged Jack is an extreme winter type relative to Tapidor_JIC and Express‐617. Fig. S2 VIN3 is rapidly induced, meaning that epigenetic dependent and independent vernalization periods are not distinguishable under our experimental conditions. Fig. S3 FLC gene sequences are sufficiently different that RNA sequencing can distinguish them. Fig. S4 FLC gene sequences for publicly available 100 bp single‐end RNA reads are sufficiently different that RNA sequencing can distinguish them. [file NPH-229-3534-s002.pdf]

New Phytologist Supporting Information

Total *FLC* transcript dynamics from divergent paralogue expression explains flowering diversity in *B. napus*

Alexander Calderwood, Andrew Lloyd, Jo Hepworth, Eleri H. Tudor, D. Marc Jones, Shannon Woodhouse, Lorelei Bilham, Catherine Chinoy, Kevin Williams, Fiona Corke, John H. Doonan, Lars Ostergaard, Judith A. Irwin, Rachel Wells, Richard J. Morris

19 November 2020

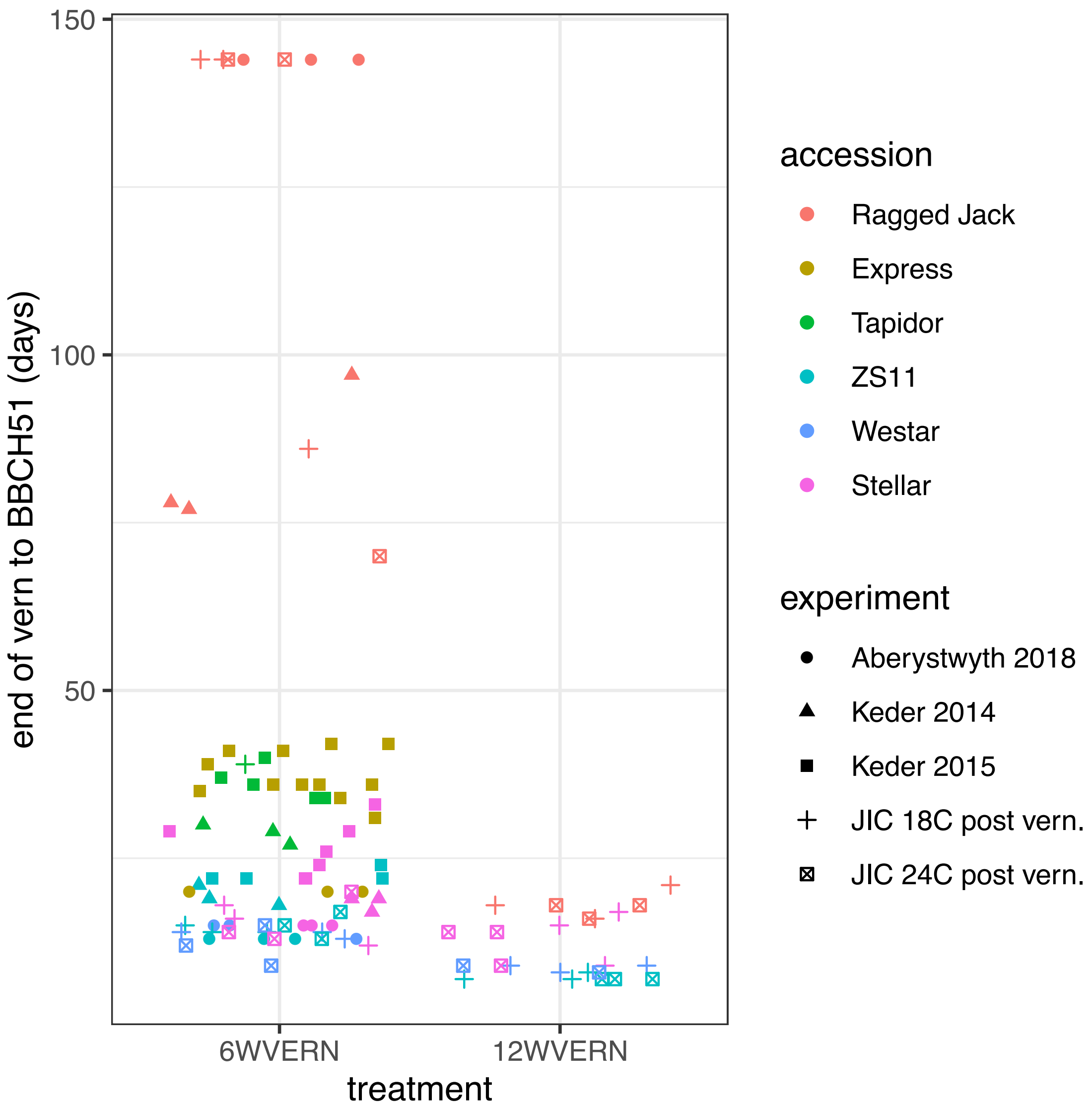

Figure S1: Ragged Jack is an extreme winter type relative to Tapidor_JIC and Express-617. Days taken after removal of plants from vernalisation to achieve BBCH51 developmental stage (buds visible). Results are plotted for individual plants across multiple trials. That Ragged Jack has a stronger vernalisation requirement that the other winter types (Express-617 and Tapidor_JIC) can be seen in the response to 6 weeks of vernalisation. Values of 144 days indicate that the individual did not flower before the end of the experiment, 144 days after vernalisation finished.

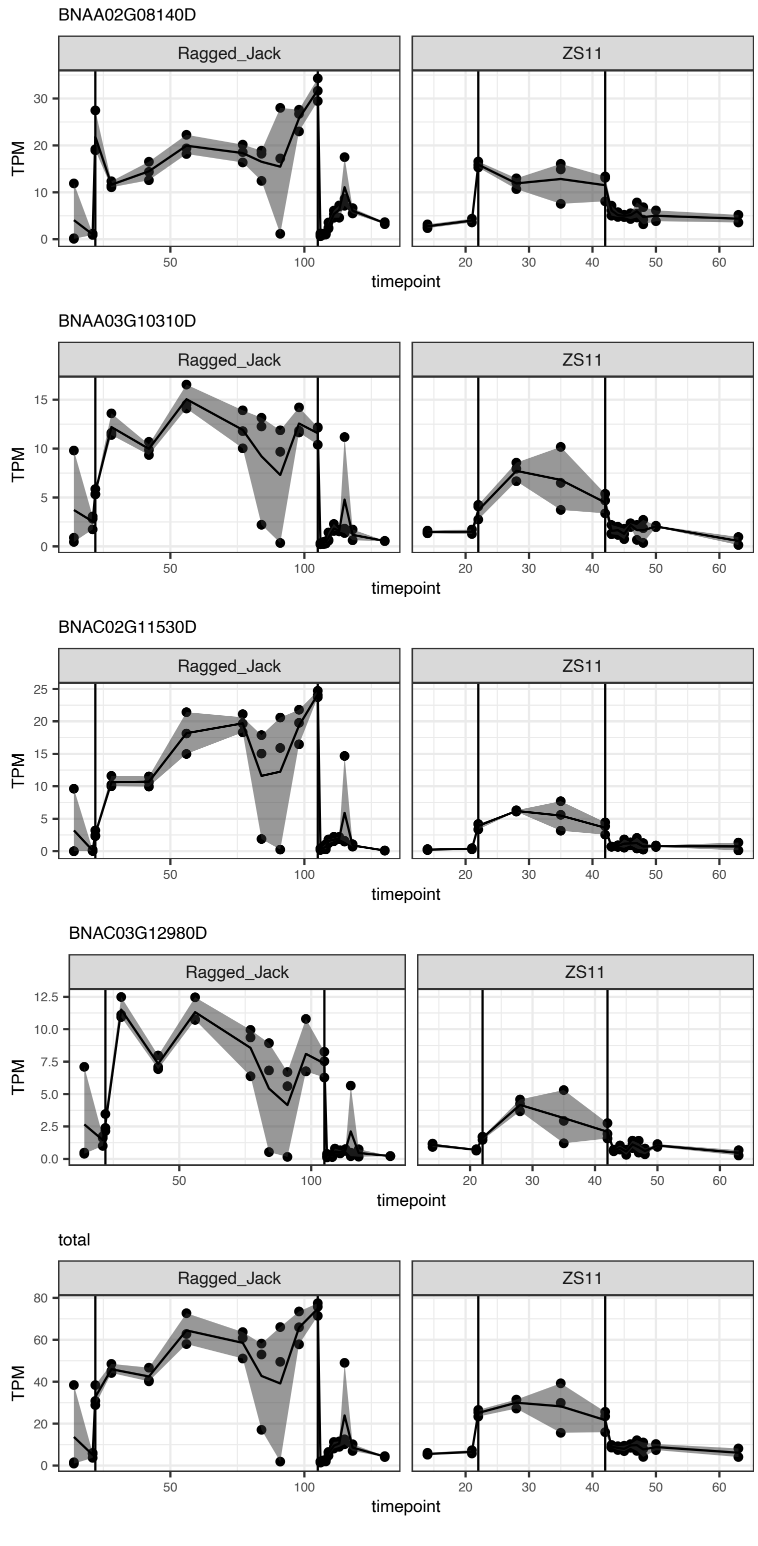

Figure S2: VIN3 is rapidly induced, meaning that epigenetic dependent and independent vernalisation periods are not distinguishable under our experimental conditions. Plots show gene expression against days from germination. vernalisation treatment at 5 °C is carried out between vertical lines. Total (summed) VIN3 expression is high at the first sampling timepoint, 1 day into vernalisation, indicating that the epigenetic dependent and independent periods of vernalisation are indistinguishable under these experimental conditions.

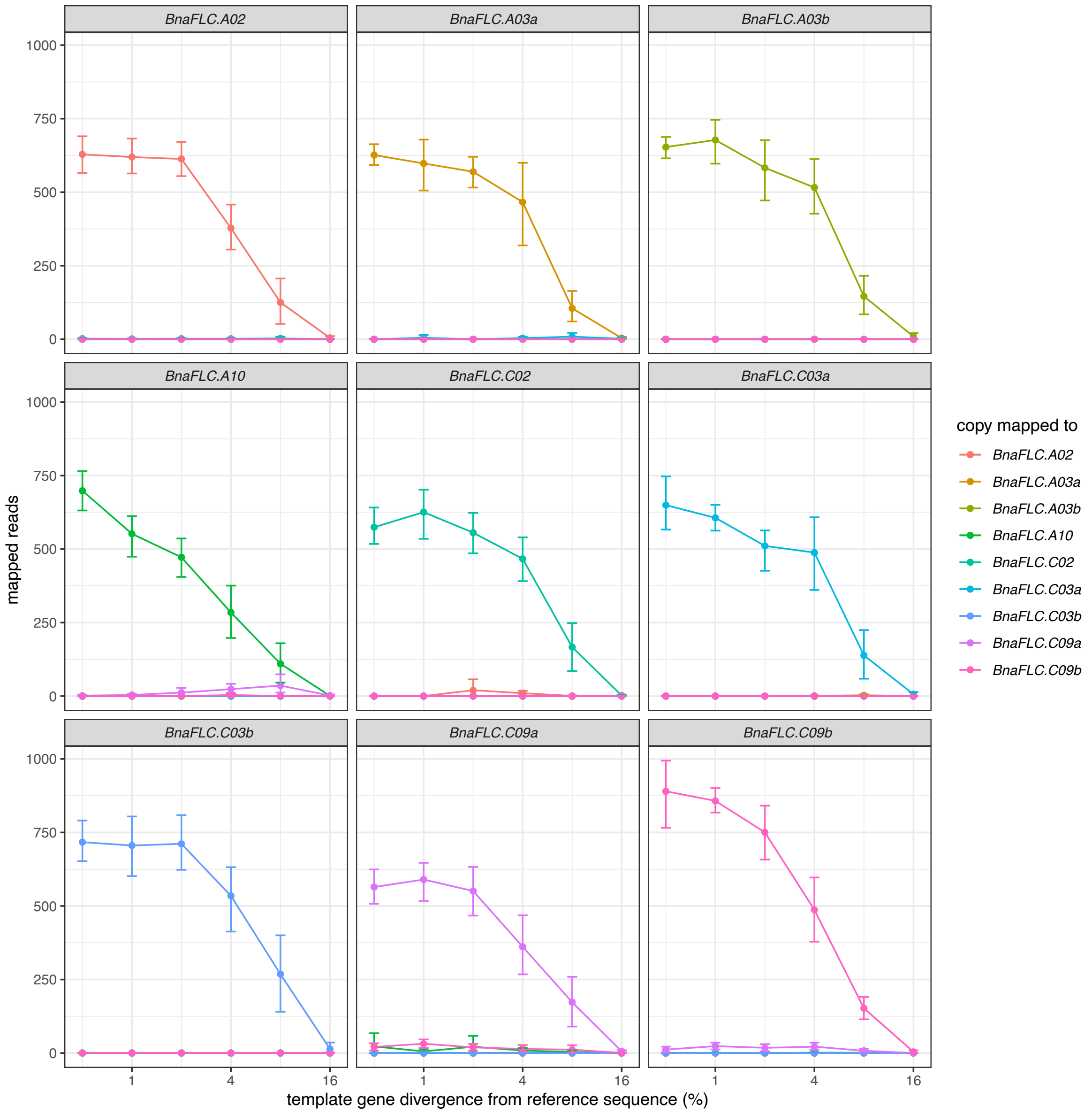

Figure S3: *FLC* gene sequences are sufficiently different that RNA-seq can distinguish them. Simulated 150 bp paired-end reads generated from each of the *FLC* gene models were aligned to the Darmor-bzh reference genomes (Chalhoub *et al.*, 2014), using the same alignment pipeline as for the real data. For each generative template sequence (facets), the number of reads mapping to each of the *FLC* gene models are plotted (colours). Divergence between the template sequence used to generate the reads, and the reference sequence of the "correct" *FLC* paralogue in the reference sequence aligned against was also considered (x-axis). For each parameter combination, 10 independent simulations were run, the mean result and estimated 95 % confidence limits are plotted. For all *FLC* paralogues, the true generative paralogue can clearly be distinguished, even for relatively high levels of divergence from the reference sequence. For comparison, the maximum experimentally identified coding sequence divergence from the reference sequence within a single accession in the RIPR panel was 1.68 %, in *BnaFLC.C03a* (**Supplemental Table 1)**.

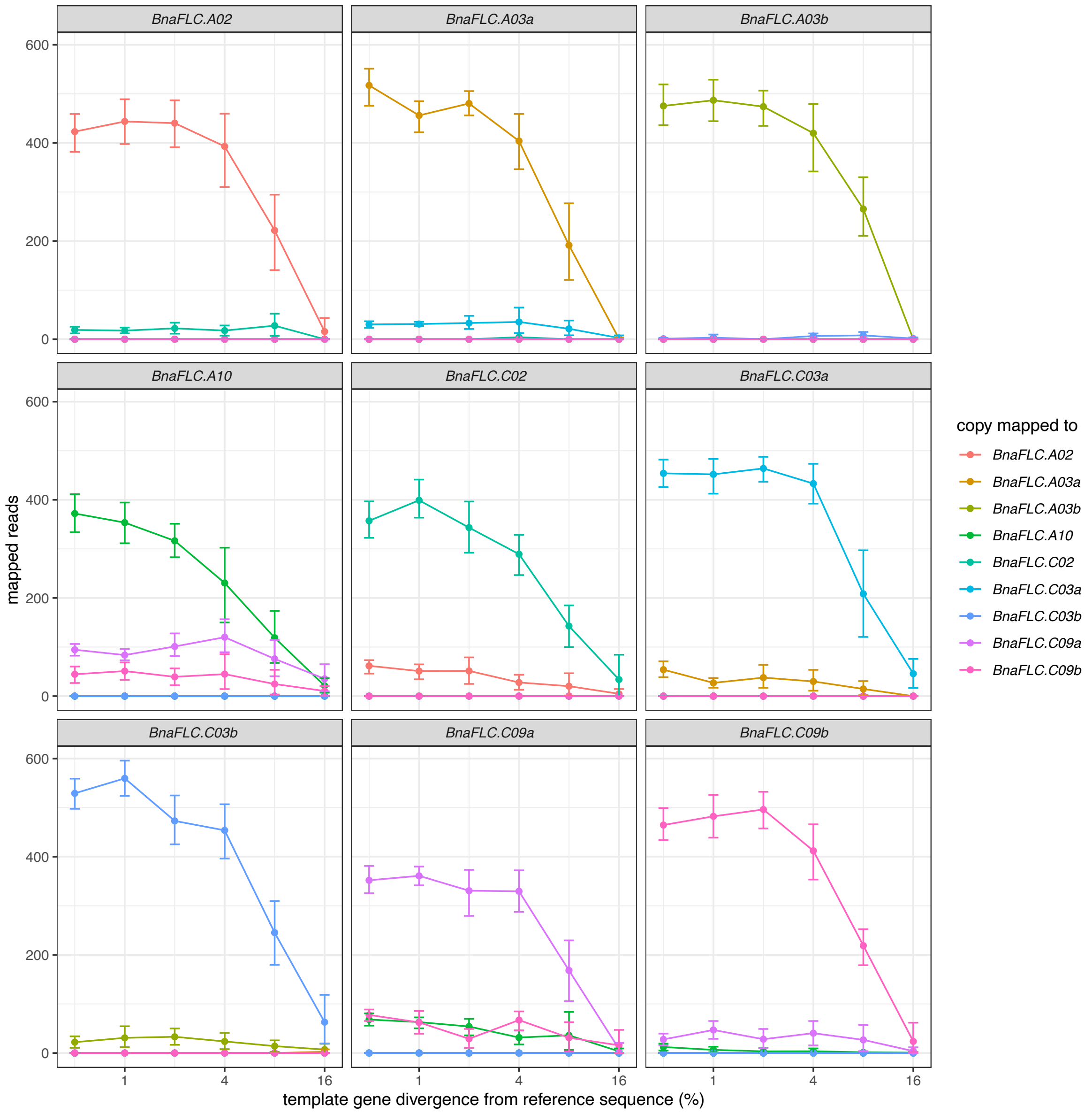

Figure S4: *FLC* gene sequences for publicly available 100 bp single-end RNA reads are sufficiently different that RNA-seq can distinguish them. Although mis-mapping rates are higher than for paired-end reads, they still map to the correct generative paralogue assuming moderate to low sequence divergence from the reference sequence. All paralogues can be clearly distinguished with some moderate mis-mapping of reads generated from *BnaFLC.A10* to *BnaFLC.C09a* and *BnaFLC.C09b*, and from *BnaFLC.C09a* to *BnaFLC.A10* and *BnaFLC.C9b*.

References

Chalhoub B, Denoeud F, Liu S, P Parkin IA, Tang H, Wang X, Chiquet J, Belcram H, Tong C, Samans B, *et al.* 2014. Early allopolyploid evolution in the post-Neolithic Brassica napus oilseed genome. *Science* **345**: 950–953.
